# Contribution of Depolarization and Repolarization Changes to J-Wave Generation and Ventricular Fibrillation in Ischemia

**DOI:** 10.3389/fphys.2020.568021

**Published:** 2020-09-30

**Authors:** Alena S. Tsvetkova, Jan E. Azarov, Olesya G. Bernikova, Alexey O. Ovechkin, Marina A. Vaykshnorayte, Marina M. Demidova, Pyotr G. Platonov

**Affiliations:** ^1^Department of Cardiac Physiology, Institute of Physiology, Komi Science Center, Ural Branch, Russian Academy of Sciences, Syktyvkar, Russia; ^2^Department of Cardiology, Clinical Sciences, Lund University, Lund, Sweden; ^3^Department of Biochemistry and Physiology, Institute of Medicine, Pitirim Sorokin Syktyvkar State University, Syktyvkar, Russia; ^4^Department of Therapy, Institute of Medicine, Pitirim Sorokin Syktyvkar State University, Syktyvkar, Russia; ^5^V. A. Almazov National Medical Research Center, Saint Petersburg, Russia; ^6^Arrhythmia Clinic, Skåne University Hospital, Lund, Sweden

**Keywords:** ischemia, J-wave, ventricular fibrillation, activation time, repolarization, ECG

## Abstract

**Background**: Activation delay in ischemic myocardium has been found to contribute to J-wave appearance and to predict ventricular fibrillation (VF) in experimental myocardial infarction. However, the role of ischemia-related repolarization abnormalities in J-wave generation remains unclear.

**Objectives**: The objective of our study was to assess a contribution of myocardial repolarization changes to J-wave generation in the body surface ECG and VF in a porcine acute myocardial infarction model.

**Methods**: In 22 anesthetized pigs, myocardial ischemia was induced by occlusion of the left anterior descending coronary artery (LAD, *n* = 14) and right coronary artery (RCA, *n* = 8). Body surface ECGs were recorded simultaneously with intramyocardial unipolar electrograms led from flexible electrodes positioned across the left ventricular (LV) wall, interventricular septum (IVS), and right ventricular (RV) wall at apical, middle and basal levels of the ventricles (a total of 48 leads). Local activation times (ATs) and activation-repolarization intervals (ARIs, differences between dV/dt maximum during T-wave and dV/dt minimum during QRS) were measured.

**Results**: J-waves appeared in left precordial leads (in 11 out of 14 animals with LAD occlusion) and right precordial leads (in six out of eight animals with RCA occlusion). During ischemic exposure, ATs prolonged, and the activation delay was associated with J-wave development (OR = 1.108 95% CI 1.072–1.144; *p* < 0.001) and VF incidence (OR = 1.039 95% CI 1.008–1.072; *p* = 0.015). ARIs shortened in the ischemic regions (in the IVS under LAD-occlusion and the lateral RV base under RCA-occlusion). The difference between maximal ARI in normal zones and ARI in the ischemic zones (ΔARI) was associated with J-wave appearance (OR = 1.025 95% CI 1.016–1.033, *p* < 0.001) independently of AT delay in multivariate logistic regression analysis.

**Conclusions**: Both AT delay and increase of ΔARIs contributed to the development of J-wave in body surface ECG. However, only AT delay was associated with VF occurrence.

## Introduction

The presence of J-wave in ECG once considered a benign phenomenon (Mehta et al., 1999) was subsequently admitted to be a predictor of idiopathic malignant ventricular tachyarrhythmias (Haissaguerre et al., 2008). J-wave appearance was also found to suggest an increased risk for ventricular tachycardia or fibrillation in acute myocardial infarction (Jastrzebski and Kukla, 2009; Naruse et al., 2012; Sato et al., 2012). During dynamic ECG observation in an experimental acute myocardial infarction model, development of J-waves preceded episodes of ventricular fibrillation (VF; Demidova et al., 2014).

Explanation for J-wave generation remains equivocal. An alternative name for this phenomenon, an early repolarization pattern, suggests an essential role of certain repolarization distinctions in its development. Indeed, in isolated myocardial preparations J-wave was demonstrated to be a manifestation of a transmural potential difference resulting from variation in spike-and-dome morphology of action potentials across the ventricular wall (Yan and Antzelevitch, 1996). A number of agents that can increase or reduce this variation, accordingly modify manifestation of the early repolarization pattern (Antzelevitch and Yan, 2010).

Development of J-waves can be also seen in depolarization disturbances. J-wave appearance in patients with non-compact cardiomyopathy, as well as in patients with arrhythmogenic right ventricular dysplasia/cardiomyopathy (ARVD/C) characterized by changes in depolarization suggests that there are alternative, including depolarization-related mechanisms of its formation (Peters and Selbig, 2008; Caliskan et al., 2012). Myocardial ischemia presents another setting where J-wave formation can result from depolarization slowing. A conduction block-related mechanism could be responsible for J-wave augmentation with cycle length shortening in acute myocardial infarction patients (Nakayama et al., 2013, 2019). Recently, in an *in vivo* model of acute myocardial infarction, we directly demonstrated an association between activation delay in the ischemic myocardium, J-wave, and VF occurrence (Azarov et al., 2019). However, a role, if any, of repolarization abnormalities in these phenomena in ischemia has not yet been tested.

The objective of the present study was to assess an association between myocardial repolarization changes and J-wave appearance in body surface ECG and VF incidence in a porcine acute myocardial infarction model.

## Materials and Methods

Experiments were performed in 22 domestic pigs (30–45 kg body weight) purchased from a local producer. The study conformed to the *Guide for the Care and Use of Laboratory Animals, 8th Edition* published by the National Academies Press (United States) 2011, the guidelines from Directive 2010/63/EU of the European Parliament on the protection of animals used for scientific purposes and was approved by the Ethical Committee of the Institute of Physiology of the Komi Science Centre, Ural Branch of Russian Academy of Sciences. Experimental procedures were described earlier (Azarov et al., 2019). Briefly, the animals were anesthetized with zoletil (Virbac S.A., Carros, France, 10–15 mg/kg, i.m.), xylazine (Interchemie, Castenray, Netherlands, 0.5 mg/kg, i.m.), and propofol (Norbrook Laboratories Ltd., UK, 1 mg/kg, i.v.), intubated and mechanically ventilated. The heart was accessed *via* a midsternal incision.

### Data Registration

**Figure 1:**
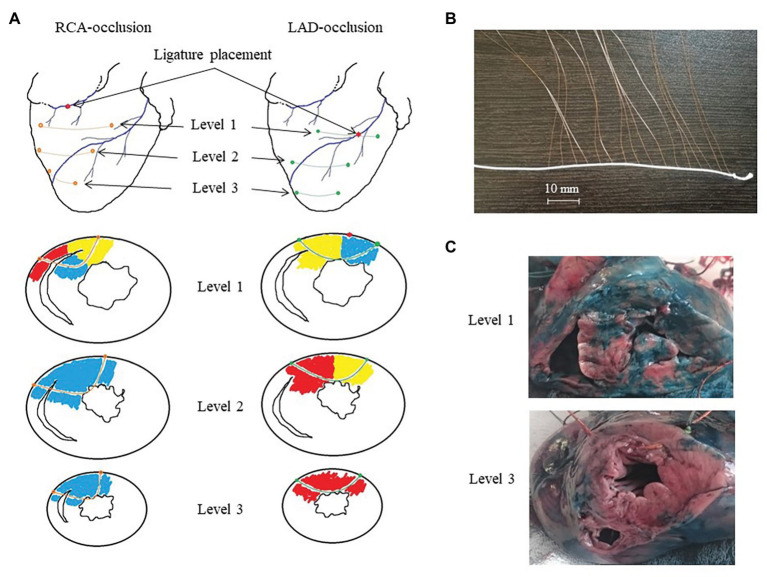
Intramyocardial recordings with flexible plunge electrodes. Panel **A**: scheme of ligature and flexible plunge electrodes placement in respect to ischemic (red), border (yellow) and normal (blue) myocardium under right coronary artery (RCA)- and left anterior descending artery (LAD)-occlusions on basal (level 1), middle (level 2) and apical (level 3) levels. Panel **B**: a photograph of a flexible plunge electrode. See an electrode filament (white thread), lead cooper wires fixed on the thread with knots with insulation removed at the knots (lead terminals). The interelectrode spacing was 5.0 mm for the basal and middle levels and 3.5 mm for the apical level. Panel **C**: photographs of porcine heart cross-cuts after the experiment with LAD occlusion after staining with Evans blue dye at the basal (level 1) and apical (level 3) levels. See mostly stained myocardium (except posterior IVS portion) at the level 1 and unstained (ischemic) myocardium at the level 3. Also, see a visible electrode filament passing through the anterior wall at the level 3 photograph.

Coronary occlusion was induced by ligation of the left anterior descending coronary artery (LAD, *n* = 14) or right coronary artery (RCA, *n* = 8). Three flexible plunge electrodes (16 lead terminals each) were placed in the anterior portion of the left ventricle (LV), interventricular septum (IVS), and right ventricle (RV) at apical, middle, and basal levels (Figure 1). The filaments of the flexible plunge electrodes were pulled through ventricular walls with a taper point surgical needle. Positions of electrodes were selected so as to comprise both expected ischemic and expected nonischemic areas (Figure 1, panel A). The electrodes were fabricated with isolated 70-μm copper wires, fixed with a knot on a 0.8-mm vicryl filament. Lead terminals on the electrode filament were equally spaced, with an interelectrode distance of 5.0 mm (electrode filament length 75 mm) for the basal and middle levels and 3.5 mm (electrode filament length 52.5 mm) for the apical level (Figure 1, panel B).

**Figure 2:**
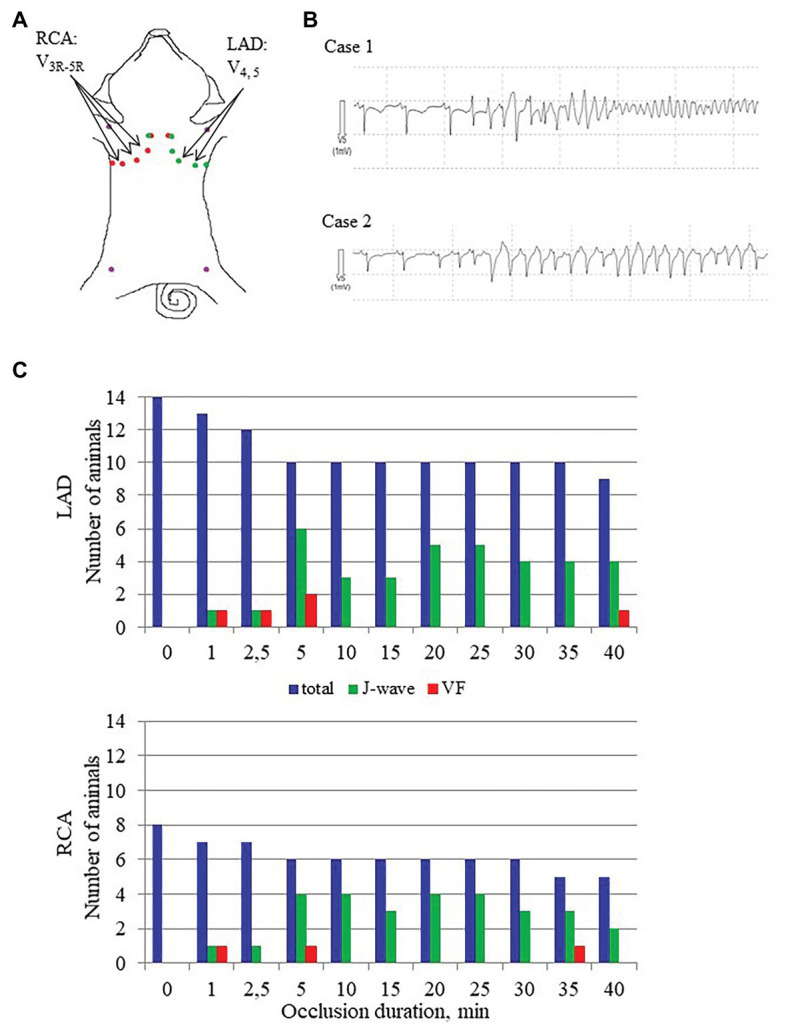
J-wave and VF incidence. Panel **A** shows distribution of body surface leads for ECG recording in conditions of LAD and RCA occlusions. Arrows indicate leads where J-waves were consistently observed in LAD and RCA occlusions. Panel **B** shows typical cases of malignant ventricular tachyarrhythmias development *via* polymorphic tachycardia (case 1), which sometimes started with irregular beats with similar QRS morphology (case 2). Panel **C** shows J-wave and ventricular fibrillation (VF) incidence during LAD (top) and RCA (bottom) occlusions. The height of the bars corresponds to numbers of animals having J-waves in ECG, numbers of animals experiencing VF at a given time-point along with a total number of animals under observation by a given time-point. Note that the total number of animals decrease as the animals were euthanized after development of VF.

**Figure 3:**
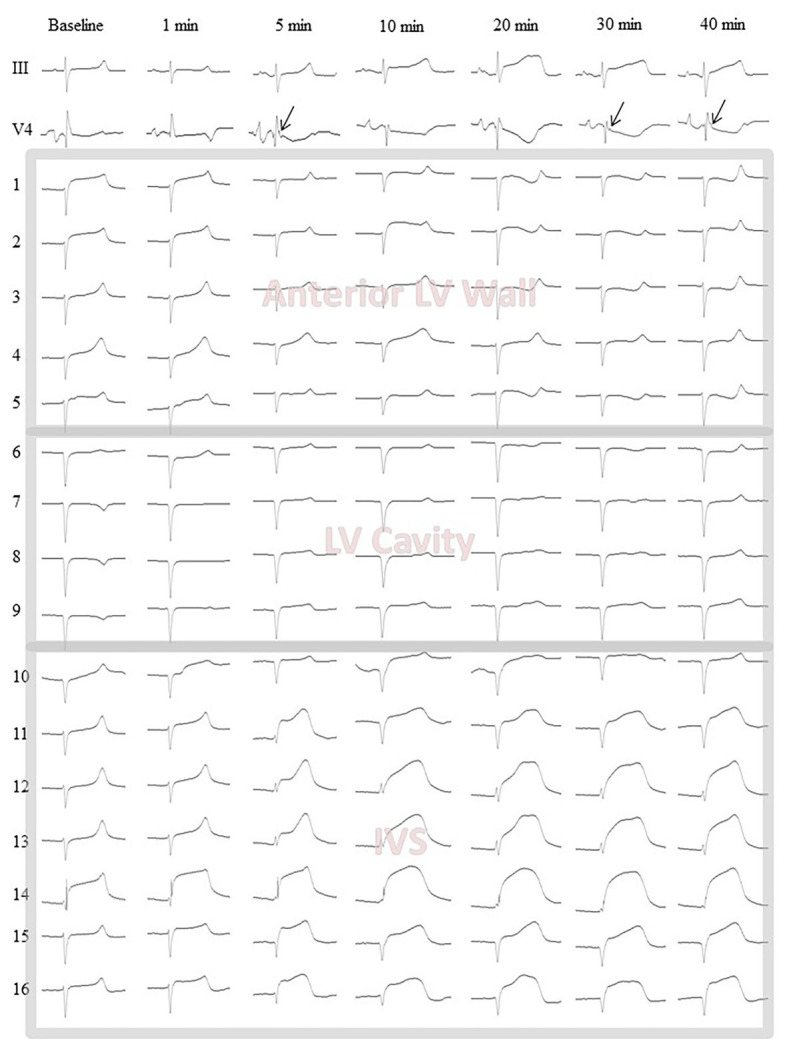
Representative body surface lead III and V4 electrocardiograms along with intramyocardial electrograms (1–16) from the middle level flexible plunge electrode in baseline and during LAD occlusion. Arrows indicate J-waves arising during occlusion in the lead V4. Leads 1–4 are located in the LV anterior wall from epicardium (1) to endocardium (4). See slight ST-segment elevation at baseline confirming a contact of the leads with myocardial tissue. Lead 5 is located at the very endocardial surface of the LV. See unstable signals (ST-segment) at baseline and 1 min likely due to mechanical artifacts caused by moving endocardial structures. Leads 6–9 are located in the LV cavity. See QS waveform of the QRS complexes and absence of dynamics during occlusion. These signals are subject to exclusion at a data processing step. Lead 10 is located at the very left endocardial surface of interventricular septum (IVS). See features similar to those of the lead five. Leads 11–16 are located in the IVS subject to ischemic insult. See dynamical changes of the QRST complex during occlusion. The lead 14 demonstrates significant ST-segment elevation at baseline due to mechanical injury. However, the ischemia-related changes are also visible in the lead 14.

After electrode and ligature placement, the chest was reclosed, and the heart was allowed to stabilize for 30 min. Recordings were done at baseline and at 1, 2.5, 5, 10, 15, 20, 25, 30, 35, and 40 min of coronary occlusion. Unipolar intramyocardial electrograms were recorded in parallel with 12 standard ECG leads and additional right precordial leads (Figure 2, panel A) by means of a custom-designed system (16 bits; bandwidth 0.05–1,000 Hz; sampling rate 4,000 Hz). Figure 3 gives an example of raw signals recorded in baseline and during LAD occlusion from the lead terminals located on the same electrode filament passing through the ventricular walls and cavities at the middle level.

The animals were euthanized under deep anesthesia by an intravenous potassium chloride injection either at the end of ischemic episode or immediately after VF development. The hearts were excised, and localization of ischemic zone (area at risk) was assessed by staining with Evans blue dye (Sigma-Aldrich GmbH, Germany), which was injected into the coronary arteries with LAD or RCA ligature tightened (Figure 1, panel C). Depths and positions of the electrodes in each heart were determined by cutting the stained heart at three levels parallel to planes of introduction of the electrode filaments. The leads located in the cavities of the ventricles and/or in the pericardial cavity were excluded from analysis.

### Data Processing

In each intramyocardial lead, local activation time (AT) and end of repolarization time (RT) were measured from the body surface QRS onset to instants of a dV/dt minimum during QRS-complex and dV/dt maximum during T-wave, respectively; activation-repolarization interval (ARI) was calculated as a difference between RT and AT (Millar et al., 1985; Haws and Lux, 1990; Coronel et al., 2006). J wave was considered present in the body surface ECGs, if it was manifested in at least two contiguous leads as notching or slurring of terminal R-wave below 50% of its height (Macfarlane et al., 2015).

A major challenge of the present study was that in the setting *in vivo* we could not utilize classical measurements of action potentials in a way performed by Yan and Antzelevitch (1996). We approached this problem as follows. A “repolarization-related” electromotive force responsible for J-wave generation is expected to develop as a difference in plateau potentials between normal and ischemic regions. The cause of this difference is apparently a drastic change in action potential morphology in the ischemic region (often referred to as “triangulation”). We assumed that the difference in the plateau potentials between the normal and ischemic regions could be reasonably estimated as a difference in local repolarization durations between these regions, which can be obtained from intramyocardial unipolar electrograms. Thus, to meet the stated objective, we tested as potential predictors a maximal AT value throughout all intramyocardial leads (AT max) and a difference between the maximal ARI throughout all intramyocardial leads and the average ARI observed in the ischemic region (ΔARI).

### Statistical Analysis

Data are expressed as medians and interquartile intervals. Statistical analysis was performed with the SPSS package (IBM SPSS Statistics 23). According to the Kolmogorov-Smirnov normality test, either parametric (two-way ANOVA with Dunnett *post-hoc* test) or nonparametric (Wilcoxon and Fridman tests) were used for repeated measurements. Comparisons between different groups of animals were done with Student’s t-test or with the Mann-Whitney test. Uni- and multi-variate logistic regression analyses were used to test for a possible relationship between intramural ATs and ARIs with appearance of J-waves on the surface ECG and VF. The differences were considered significant at *p* < 0.05.

## Results

### J-Wave and VF Incidence

**Figure 4:**
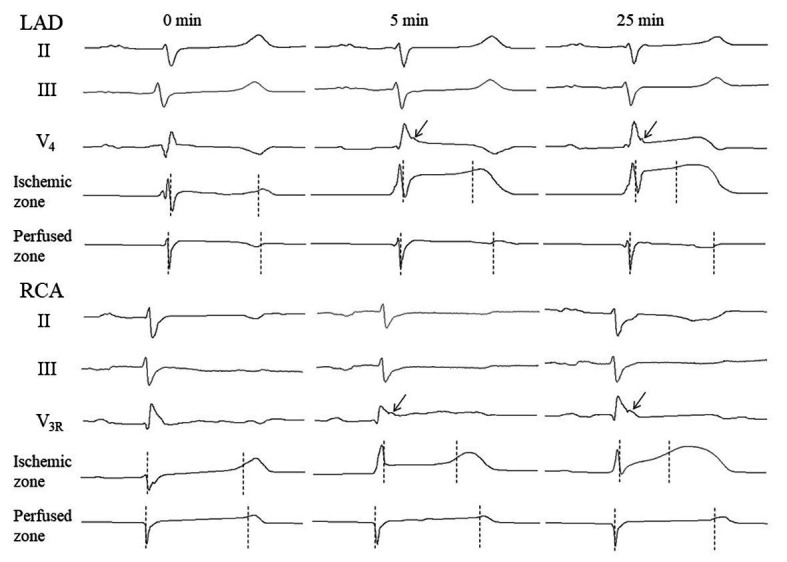
Representative ECGs and intramyocardial electrograms from perfused and ischemic regions at baseline, and at early and late periods of coronary occlusion. Vertical markers show activation time (AT) and repolarization time (RT) instants. Arrows point to J-waves. See activation-repolarization interval (ARI) shortening in the ischemic zone at the 5th and 25th min vs. ARI no changing at the same time-points in the perfused zone.

**Figure 5:**
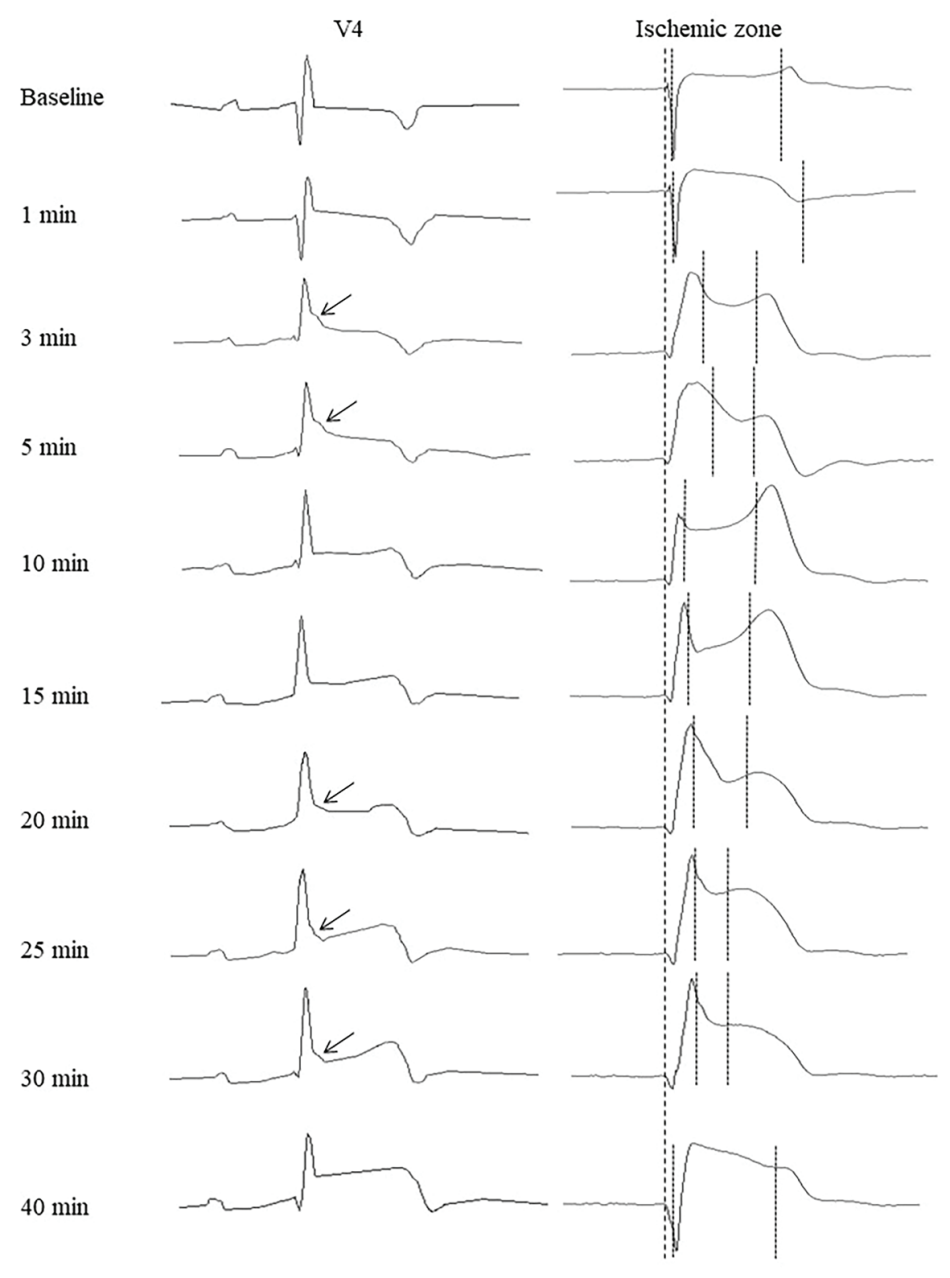
Representative precordial electrocardiograms and unipolar electrograms demonstrating J-wave formation during LAD occlusion. Arrows indicate J-waves in the lead V4 electrocardiograms. Thick dotted lines indicate a QRS onset in cardiac signals serving as a reference time-point for measurements of activation times and end of repolarization times (thin dotted lines). See correlation between J-wave development and marked prolongation of activation time and/or shortening of end of repolarization time.

The body surface ECGs demonstrated no J-wave pattern in any leads at baseline. J-waves developed approximately in three quarters of pigs (11 out of 14 in the LAD group and six out of eight in the RCA group; Figure 2). J-waves were observed either steadily (nine and four animals in LAD and RCA group, respectively) or transiently (two animals in each group) in the period after 2.5–5 min until the end of ischemic exposure. Under LAD occlusion, J-waves were mostly observed in leads V_4_–V_5_ and aVL. Under RCA occlusion, J-waves could be found in leads V1–V2 among the standard 12 leads, but mostly in additional right-sided and elevated precordial leads. Top tracings in Figure 4 give examples of J-wave formation at the 5th and 25th min of ischemic exposure. Morphological characteristics of J-waves were not constant during occlusion. There could be slur to notch transformation (Figure 4) or dynamical changes of its amplitude and duration (Figure 5). Eight pigs (five out of 14 in the LAD group and three out of eight in the RCA group) experienced VF, after which the animals were euthanized. J-wave development preceded six out of eight (75%) VF episodes but was not a significant predictor of VF (OR 1.20 95% CI 0.17–8.66, *p* = 0.857). The most common type of VF initiation (Figure 2, panel B) was through polymorphic tachycardia (case 1), which in some animals sometimes started with irregular beats with similar QRS morphology (case 2). VF developed mostly during the first 5 min of coronary occlusion (Figure 2, panel C).

### AT Dynamics

**Figure 6:**
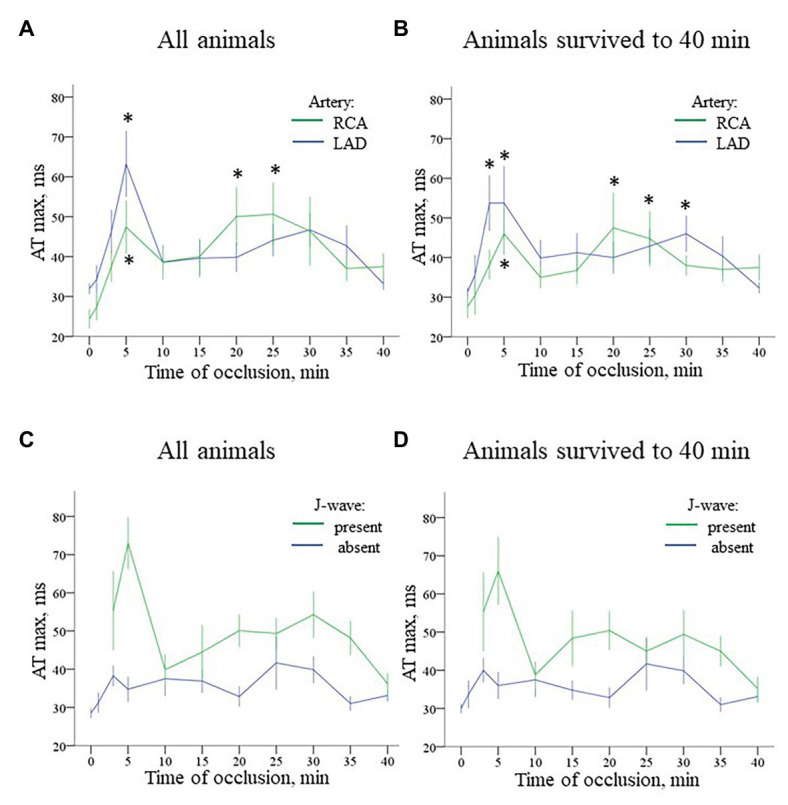
Time-course of evolution AT max (Mean ± SEM) during coronary occlusion in all animals (panels **A,C**) and in the animals that survived until the 40th min of occlusion and experiencing no ventricular fibrillation (VF; panels **B,D**). Panel **A** shows AT max changes during LAD (*n* = 14) and RCA (*n* = 8) occlusion. See quite similar dynamics with early and delayed peaks of AT prolongation in both groups. Panel **B** shows analogous AT max changes in animals that survived until the 40th min of ischemia (i.e., experiencing no VF) during LAD (*n* = 9) and RCA (*n* = 6) occlusion. See that though AT max prolongation is lower in the no-VF group, the overall dynamics of AT max changes is similar to that in all animals displayed in panel **A**. Panel **C** presents comparison of AT max for the cases with and without J-waves in the body surface ECG in the combined group (LAD and RCA occlusion together, *n* = 22). Panel **D** shows analogous data for the animals that survived until the 40th min of occlusion (*n* = 15). ^*^p < 0.05 vs. baseline (ANOVA, *post-hoc* Dunnet test).

In the baseline state, ATs were relatively uniform throughout ventricular myocardium, and AT max was usually observed in the basal regions. During ischemic exposure, AT max was found in the ischemic or border areas according to location of occlusion (LAD or RCA). The ischemic regions were a middle part of IVS and a base of the lateral RV wall in LAD and RCA groups, respectively. During occlusion of both arteries, AT max similarly increased in a biphasic manner with two maxima at approximately 5 and 20–30 min of ischemic episode. Figure 5 gives an example of the biphasic change of AT in the same lead located in the ischemic zone. It can be seen that the extent of AT prolongation is higher in the earlier phase. Similarly, AT max is longest at 3–5 min (Figure 6). In the animals that survived until the end of occlusion (it means that these animals did not experience VF), quite a similar dynamics of AT max was found, though the magnitude of maxima were lower (compare panels A,B in Figure 6). In LAD occlusion, the AT max tended to be higher than in RCA occlusion, but the differences did not reach statistical significance. Irrespectively of occlusion duration, AT max observed simultaneously with J-wave in the body surface ECG was significantly longer than AT max not associated with J-wave on surface ECG [49 (IQR 39–63) vs. 34 (IQR 31–39) ms, *p* = 0.001, respectively, Figure 6, panels C,D]. Figure 5 shows that the body surface J-wave developed when AT in the ischemic zone significantly prolonged and disappeared when AT transiently recovered. In univariate logistic regression analysis, AT max was associated with J-wave development both in LAD (OR = 1.118 95% CI 1.072–1.165; *p* < 0.001) and RCA (OR = 1.051 95% CI 1.008–1.098; *p* < 0.020) occlusions. In a whole group (LAD + RCA occlusion, *n* = 22), AT max was associated with both J-wave (OR = 1.108 95% CI 1.072–1.144; *p* < 0.001) and VF (OR = 1.039 95% CI 1.008–1.072; *p* = 0.015) incidence. In the animals that survived until the end of occlusion (nine in LAD and six in the RCA group of animals without VF), univariate analysis also showed that AT max was associated with J-wave development in LAD (OR = 1.115 95% CI 1.063–1.168; *p* < 0.001; *n* = 9), RCA (OR = 1.089 95% CI 1.014–1.171; *p* = 0.020; *n* = 6) occlusions, and in the whole No-VF group (LAD + RCA, *n* = 15; OR = 1.100 95% CI 1.062–1.139; *p* < 0.001; *n* = 15).

### ARI Dynamics

**Figure 7:**
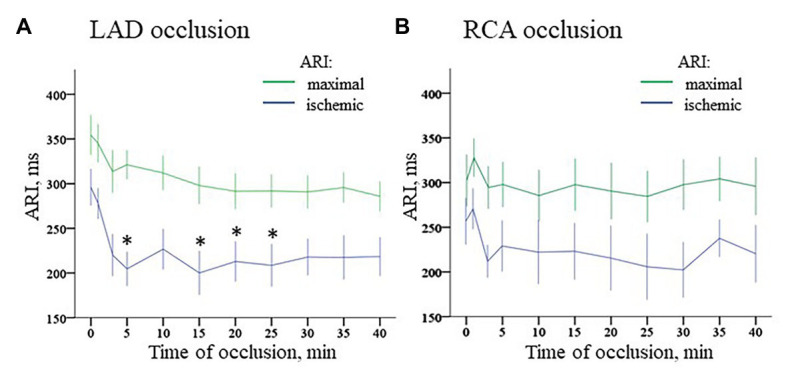
Dynamics of ischemic and maximal ARI changes during coronary occlusion. Panel **A** displays changes in ARIs in the ischemic zone (middle part of IVS) and maximal ARIs observed in the normal myocardium during LAD occlusion. Similarly, panel **B** displays changes of ARIs in the ischemic zone [base of lateral right ventricular (RV) wall] and maximal ARIs observed in the normal myocardium during RCA occlusion. ^*^p < 0.05 vs. baseline (ANOVA, *post-hoc* Dunnet test).

Durations of maximal ARIs (consistently observed in the nonischemic zones) demonstrated no significant changes in comparison with baseline in both LAD and RCA groups (Figure 7, panels A,B). By the end of ischemic exposure, significant shortening of ARIs was observed in these regions [223 (IQR 222–236) ms at 40th min of LAD occlusion vs. 307 (IQR 302–323) in baseline, *p* = 0.003; and 213 (IQR 157–287) ms at 40th min of RCA occlusion vs. 253 (IQR 186–316) ms in baseline, *p* = 0.030].

**Figure 8:**
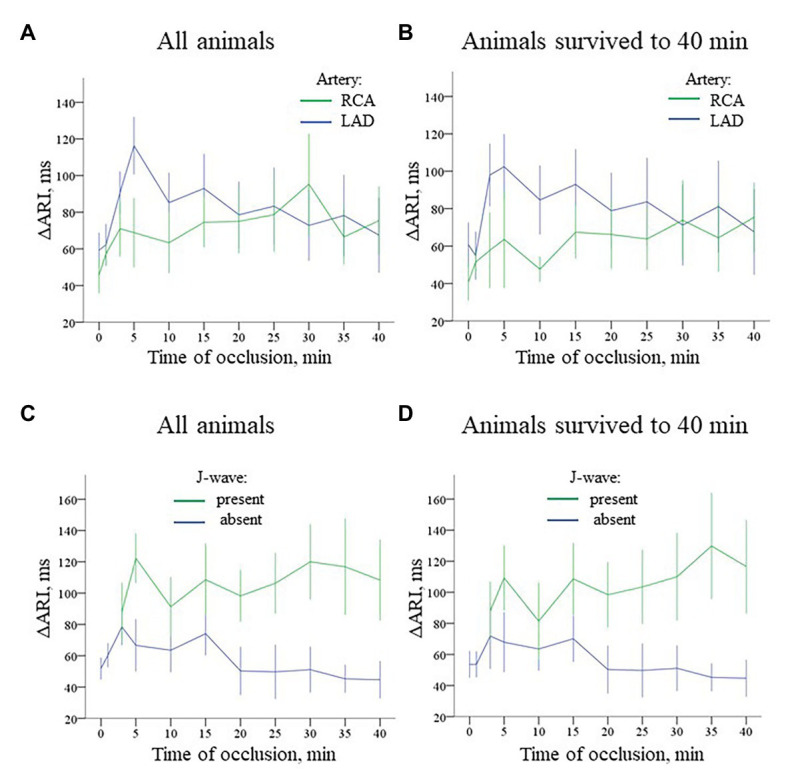
Time-course of evolution of ΔARI (Mean ± SEM) during coronary occlusion in all animals (panels **A,C**) and in the animals that survived until the 40th min of occlusion and experiencing no VF (panels **B,D**). Data are presented similarly to Figure 6. Panel **A** ΔARI changes during LAD (*n* = 14) and RCA (*n* = 8) occlusion. See different ΔARI dynamics in two groups caused by different patterns of ARI changes in the ischemic regions depicted in Figure 7. Panel **B** ΔARI changes in the animals that survived until the 40th min of ischemia (i.e., experiencing no VF) during LAD (*n* = 9) and RCA (*n* = 6) occlusion. Panel **C** Comparison of ΔARI for the cases with and without J-waves in the body surface ECG in the combined group (LAD and RCA occlusion together, *n* = 22). Panel **D** shows analogous data for the animals that survived until the 40th min of occlusion (*n* = 15).

ARI distribution in the myocardium in baseline was not uniform. Median ARIs in the RV base were shorter, than those in the LV base [253 (IQR 186–316) ms vs. 302 (259–316) ms, respectively; *p* < 0.001]. At the apical level, ARIs did not differ between RV and LV [261 (IQR 182–357) ms and 283 (212–323) ms, respectively; *p* = 0.377]. Multiple comparisons of ARIs at each time-point during ischemia vs. baseline showed different dynamics in the LAD and RCA groups (Figure 7, panels A,B). The former demonstrated significant shortening mostly at the beginning of occlusion. In contrast, RCA ligation induced less pronounced changes in the affected region. At the very beginning, there was even moderate ARI increase in several animals. However, ischemia-related shortening progressively increased up to the end of RCA occlusion. As a result, ΔARI developed during the ischemic episodes. As displayed in Figure 8, panel A, dynamics of ΔARI differed for two occlusion sites. In LAD occlusion, a dramatic increase of ΔARI was observed during first 5 min with subsequent partial restoration till the end of ischemic exposure. In RCA occlusion, ΔARI progressively increased during the whole episode, but the extent of ΔARI increase was less than in LAD occlusion.

Irrespectively of the infarct-related artery, ∆ARI was significantly higher in the presence of J-waves in the body surface ECGs [97 (IQR 71–155) vs. 49 (IQR 29–97) ms, *p* = 0.001, respectively, Figure 8, panel C]. Figure 5 shows that J-wave development corresponded to the shortest ARIs in the ischemic zone resulting in increased ΔARI. In univariate logistic regression analysis, ΔARI was associated with J-wave development both in LAD (OR = 1.029 95% CI 1.018–1.040; *p* < 0.001, *n* = 14) and RCA (OR = 1.019 95% CI 1.005–1.034; *p* = 0.008, *n* = 8) groups. In the combined group (LAD + RCA), ΔARI was also associated with J-wave occurrence (OR = 1.025 95% CI 1.016–1.033, *p* < 0.001, *n* = 22) but not with VF (OR = 1.007 95% CI 0.994–1.020, *p* = 0.313). The similar dynamics of ΔARI was shown in the animals survived the entire 40 min period of occlusion (Figure 8, panels B,D). In these animals, ΔARI was also associated with J-wave appearance in LAD (OR = 1.030 95% CI 1.017–1.043; *p* < 0.001, *n* = 9), RCA (OR = 1.018 95% CI 1.004–1.033; *p* = 0.013, *n* = 6), and combined LAD + RCA (OR = 1.024 95% CI 1.015–1.032, *p* < 0.001, *n* = 15) groups.

**Figure 9:**
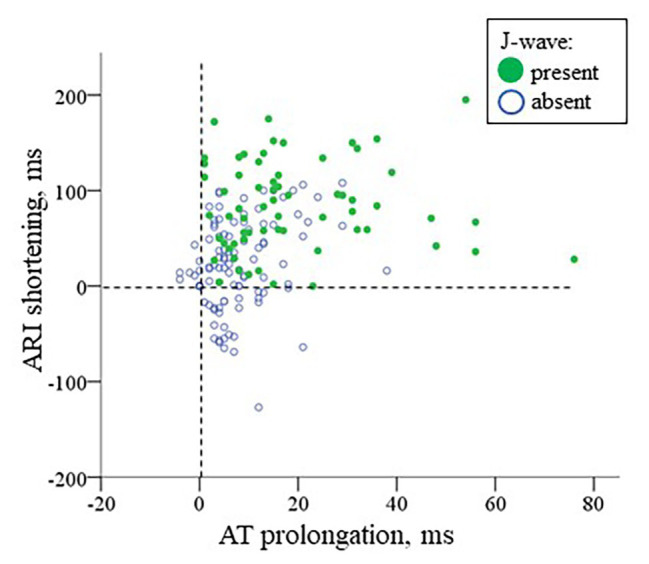
A scatter plot for AT prolongation vs. ARI shortening in the ischemic region in respect to baseline values for cases with and without J-waves in the body surface ECG in the combined group (LAD and RCA occlusion together, *n* = 22). The negative values indicate AT shortening or ARI prolongation, respectively.

In multivariate logistic regression analysis, it was shown that both AT max and ΔARI (i.e., the activation delay in the ischemic zone and the difference between the maximal ARI and ARI in the ischemic zone) were independent predictors (OR = 1.078 95% CI 1.040–1.118, *p* < 0.001 and OR = 1.016 95% CI 1.007–1.025, *p* = 0.001, respectively, *n* = 15) of J-wave appearance on ECG during coronary occlusion of both localizations. A scatter plot in Figure 9 displays a relationship between AT prolongation and ARI shortening in the ischemic zone in respect to baseline (time-point 0) and demonstrates that J-waves were not observed in cases when ARI prolonged in the affected region (Chi-square 16.7, *p* < 0.001). These cases are indicated by blue circles in a domain of negative ARI shortening (that means ARI prolongation). Collectively, these findings suggest independent contribution to J-wave generation from depolarization and repolarization changes.

## Discussion

In the present study, we demonstrated that in experimental ischemia J-wave development was associated with the activation delay and increased difference in repolarization durations between normal and ischemic regions. These relationships were similar in conditions where either left or right ventricular myocardium was predominantly affected, except for the expected differences in the leads where J-waves were observed. The activation delay, but not repolarization abnormalities was associated with VF.

Appearance of J-waves in the body surface ECG is considered as a marker of arrhythmic risk. Despite a high interest, the origin of this phenomenon observed in quite different conditions remains unclear, and both depolarization- and repolarization-related mechanisms are under discussion (Haïssaguerre et al., 2019). Myocardial ischemia provides a setting with increased risk for fatal ventricular tachyarrhythmia caused by severe electrophysiological changes in the myocardium. These changes can be loosely categorized (similarly to the mechanisms of J-wave generation) as related to depolarization and repolarization disorders. The former basically concerns conduction slowing (Kleber et al., 1986) caused by inactivation of sodium channels (Kleber and Rudy, 2004) and gap junctions uncoupling (De Groot and Coronel, 2004), and the latter is largely concerned with action potential duration shortening induced largely by activation of I_K(ATP)_ current among other causes (Shaw and Rudy, 1997; Carmeliet, 1999).

Theoretically, generation of J-wave should be based on a potential difference arising between ventricular regions shortly after the QRS-complex with the region proximal to the lead to be positively charged (i.e., less depolarized). This condition is met under ischemia when the activation wave is slowly spreading toward a positive lead terminal across the ischemic area. Similarly, J-wave appeared under inhibition of sodium current (Meijborg et al., 2016). In the previous study (Azarov et al., 2019), we directly showed an association between activation delay in the ischemic myocardium and J-wave development in the precordial leads. Clinical data (Nakayama et al., 2013, 2019) also suggest that conduction delay facilitates formation of J-waves in patients with myocardial ischemia.

Similarly to the condition of delayed depolarization, less depolarized myocardium can be found in conditions of accelerated phase 1 repolarization. Such mechanism of J-wave generation was demonstrated in ventricular wedge preparations when intrinsic transmural difference in spike-and-dome morphology of action potentials is increased due to variation in I_to_ current density (Yan and Antzelevitch, 1996; Koncz et al., 2014). In the ischemic cardiac tissue, action potential repolarization shortens and loses a plateau phase (Shaw and Rudy, 1997; Carmeliet, 1999). Apparent manifestation of these changes resembles the phase 1 acceleration and together with the delayed activation can produce overlapping electric fields resulting in J-waves (Bacharova et al., 2013; Bacharova and Halkias, 2016). However, our present study is, to our knowledge, the first to report a direct association between ischemia-induced shortening of repolarization and J-wave formation in the body surface ECG.

The repolarization-related predictor of J-wave development used in our present study was the difference between the “ischemic” (i.e., shortest) ARI and ARI in the normal myocardium. Using this parameter, we assumed that the greater was this duration difference, the greater was the potential difference between these regions, and therefore the greater was the electric field responsible for J-wave development. Interestingly, we observed much less ΔARI in RCA occlusion, whereas dynamics of ATs were quite similar in the RV and LV ischemic areas under RCA and LAD occlusion, respectively. Though the different repolarization responses to ischemia cannot be explained directly by the data obtained, this observation is consistent with the findings of Pandit et al. (2011) demonstrating a larger I_K(ATP)_ current in the LV as compared to the RV. Moreover, ARIs in the RV base were shorter in baseline and probably had less repolarization reserve. Anyway, the ΔARI parameter was an independent predictor of J-wave appearance both in uni- and multi-variate (together with activation delay) analysis, both in LAD- and RCA-occlusion groups separately as well as in combination.

The interest to J-waves as a distinct ECG phenomenon is well-understood taking into account its potential use as a predictor of fatal ventricular arrhythmias. However, it was pointed out that not every ever observed J-wave necessarily predicts ventricular fibrillation or sudden cardiac death (Bourier et al., 2018; Demidova et al., 2019). Our previous (Azarov et al., 2019) and present studies demonstrated that both repolarization- and depolarization-related mechanisms are involved in J-wave generation in the ischemic conditions. However, only activation delay is associated with VF incidence. Furthermore, a threshold for activation delay needed for VF development is higher than for J-wave generation. These data imply that only deeply affected individuals demonstrating J-waves would suffer from VF.

Thus, in the open chest model of myocardial infarction, repolarization abnormalities expressed as a difference in ARIs between the normal and ischemic myocardium contributed to J-waves formation together with activation delay, but did not predict VF occurrence, which was associated only with activation slowing.

### Limitations

Estimation of action potential duration as ARI obtained from the *in vivo* recorded myocardial electrograms can be considered as reliably validated (Millar et al., 1985; Haws and Lux, 1990; Coronel et al., 2006). However, the action potential phase 1 repolarization supposedly related to generation of J-wave occurs “within an ARI” and could not be recorded directly. To evaluate repolarization abnormalities in this period, we assumed that the well-known triangularization of the action potential in the ischemic myocardium is expressed in changes of ARIs. This approach presents a main limitation for this work and warrants cautious interpretation of the results. However, the data obtained in the ischemic porcine myocardium (Coronel et al., 1992) suggest a correlation between changes in action potential shape and duration, whereas the latter could be estimated from the unipolar intramyocardial electrograms.

Ischemic conditions impose additional limitations on interpretation of measured ARIs since morphology of action potentials is significantly altered under ischemia. However, ARI measurements and analysis are still feasible in such settings (Ejima et al., 1998). Moreover, we did not attempt to determine exact action potential durations which might have been underestimated in assessments with the aid of ARI in hypoxic myocardium. Our goal was only to attain an indirect evaluation of changes of action potential morphology supposedly responsible for J-wave generation, and such evaluation, if done cautiously, can be considered reasonable.

## Data Availability Statement

The raw data supporting the conclusions of this article will be made available by the authors, without undue reservation.

## Ethics Statement

The animal study was reviewed and approved by the Ethical Committee of the Institute of Physiology of the Komi Science Centre, Ural Branch of Russian Academy of Sciences.

## Author Contributions

AT, JA, OB, AO, MD, and PP contributed to the design. AT, JA, OB, AO, MV, and MD contributed to the experiments. AT, JA, AO, and MV contributed to the data analysis. AT and JA drafted the article. All authors contributed to the article and approved the submitted version.

### Conflict of Interest

The authors declare that the research was conducted in the absence of any commercial or financial relationships that could be construed as a potential conflict of interest.
